# *Hypoxia-inducible factor 3A* gene expression and methylation in adipose tissue is related to adipose tissue dysfunction

**DOI:** 10.1038/srep27969

**Published:** 2016-06-27

**Authors:** Susanne Pfeiffer, Jacqueline Krüger, Anna Maierhofer, Yvonne Böttcher, Nora Klöting, Nady El Hajj, Dorit Schleinitz, Michael R. Schön, Arne Dietrich, Mathias Fasshauer, Tobias Lohmann, Miriam Dreßler, Michael Stumvoll, Thomas Haaf, Matthias Blüher, Peter Kovacs

**Affiliations:** 1 Department of Medicine, Dermatology und Neurology, Department of Endocrinology und Nephrology, University of Leipzig, Leipzig, Germany; 2 Leipzig University Medical Center, IFB AdiposityDiseases, University of Leipzig, Leipzig, Germany; 3 Institute of Human Genetics, University of Würzburg, Würzburg, Germany; 4 Clinic of Visceral Surgery, Städtisches Klinikum Karlsruhe, Karlsruhe, Germany; 5 Department of Surgery, University of Leipzig, Leipzig, Germany; 6 Municipal Clinic Dresden-Neustadt, Dresden, Germany

## Abstract

Recently, a genome-wide analysis identified DNA methylation of the *HIF3A* (*hypoxia-inducible factor 3A*) as strongest correlate of BMI. Here we tested the hypothesis that *HIF3A* mRNA expression and CpG-sites methylation in adipose tissue (AT) and genetic variants in *HIF3A* are related to parameters of AT distribution and function. In paired samples of subcutaneous AT (SAT) and visceral AT (VAT) from 603 individuals, we measured *HIF3A* mRNA expression and analyzed its correlation with obesity and related traits. In subgroups of individuals, we investigated the effects on *HIF3A* genetic variants on its AT expression (N = 603) and methylation of CpG-sites (N = 87). *HIF3A* expression was significantly higher in SAT compared to VAT and correlated with obesity and parameters of AT dysfunction (including CRP and leucocytes count). *HIF3A* methylation at cg22891070 was significantly higher in VAT compared to SAT and correlated with BMI, abdominal SAT and VAT area. Rs8102595 showed a nominal significant association with AT *HIF3A* methylation levels as well as with obesity and fat distribution. *HIF3A* expression and methylation in AT are fat depot specific, related to obesity and AT dysfunction. Our data support the hypothesis that HIF pathways may play an important role in the development of AT dysfunction in obesity.

Obesity and its associated comorbidities constitute an evolving health burden worldwide^1^. Obesity is closely related to chronic inflammation in adipose tissue, liver and skeletal muscle^2^, which may contribute to chronic systemic inflammation, insulin resistance, and deterioration in glucose and lipid metabolism^3^. Upon weight gain, adipocyte hypertrophy may lead to hypoxia in adipose tissue which is considered as a causative factor in adipose tissue dysfunction^4^,^5^,^6^,^7^. It has been recently shown that adipose tissue expression of *hypoxia inducible factor (HIF) 1A* (*HIF1A*) increases in mice exposed to high fat diet^4^. In states of relative adipose tissue hypoxia, induction of HIF1α^5^,^6^ stimulates accumulation of macrophages in adipose tissue^4^,^7^ and the production of adipocyte-derived pro-inflammatory cytokines. HIFs are heterodimeric transcription factors that mediate hypoxia response in various tissues^6^. They consist of an oxygen-labile α-subunit and a constitutively expressed β-subunit. Three existing isoforms of the α-subunit, HIF1α, HIF2α and HIF3α, allow the formation of transcription factors with different functions upon dimerizing with HIFβ. Multiple isoforms of HIF3α exist^8^. HIF3α is capable of activating certain target genes independent or in collaboration with HIF1α, suggesting a role of HIF3α in glucose and amino acid metabolism, apoptosis, proteolysis, p53 signaling and PPAR signaling. In addition, HIF3α has been shown to play a role in adipocyte differentiation^9^,^10^.

Recent genome-wide analysis of DNA methylation in whole blood and human adipose tissue revealed an association of methylation at three CpG sites in intron 1 of *HIF3A* with BMI^11^,^12^,^13^. In addition, two single nucleotide polymorphisms (SNPs) rs8102595 and rs3826795, have been shown to be associated with methylation at these sites, yet to be independent of BMI^11^. The strong relationship of *HIF3A* methylation and obesity was also shown in neonates^14^. Furthermore, gene-diet interactions between the methylation-associated SNP rs3826795 and vitamin B intake were recently reported, providing a potential causal link between the epigenetic status and obesity^15^.

Further investigation of the relationship between HIFs and development of obesity-associated comorbidities might reveal important insights in pathophysiological processes concerning AT inflammation and/or insulin resistance in the etiology of obesity related metabolic diseases. We therefore tested the hypothesis that expression of *HIF3A* in human subcutaneous and visceral adipose tissue is related to obesity, parameters of fat distribution and adipose tissue function. We further assessed the relationship between the AT expression, genetic variation (rs8102595 and rs3826795) and methylation of CpG-sites in *HIF3A*.

## Material and Methods

### Study participants

A total of 288 Caucasian men and 577 women were included in the study (Table 1). According to the ADA criteria, 343 subject were diagnosed with type 2 diabetes (T2D) and 484 had normal glucose tolerance (NGT)^16^. Paired samples of visceral adipose tissue and subcutaneous adipose tissue were obtained from 603 individuals following open abdominal surgery for gastric banding, cholecystectomy, weight reduction surgery, abdominal injuries or explorative laparotomy. Patients with end-stage malignant diseases were excluded from the study. All adipose tissue samples were frozen immediately in liquid nitrogen after explantation and stored at −80 °C. Six-hundred and three subjects (mean age 50 ± 14 years, mean BMI 43.6 ± 13.0 kg/m^2^) were included into adipose tissue *HIF3A* mRNA expression analysis. DNA methylation analysis was performed in a subgroup of 87 subjects (mean age 58 ± 15 years, mean BMI 32.9 ± 12.7 kg/m^2^). Genotyping was done in 548 individuals overlapping with adipose tissue biopsy donors (mean age 50 ± 14 years, mean BMI 34.6 ± 13.6 kg/m^2^).

Phenotypic characterization including anthropometric measurements, body fat analysis (bioimpedance analyses or dual-energy X-ray absorptiometry) and metabolic parameters such as fasting plasma glucose and insulin, a 75-g oral glucose tolerance test (oGTT), HbA1c, lipoprotein-, triglyceride-, free fatty acid- and adipokine serum concentrations was performed as previously described^17^,^18^. Measurement of abdominal visceral and subcutaneous fat areas (N = 245) was performed using computed tomography (CT) or MRI scans. All subjects had a stable weight, defined as the absence of fluctuations of >2% of body weight for at least 3 months before surgery. In addition, adipocytes and cells of the stromal vascular fraction were isolated from adipose tissue samples of 35 subjects (18 men, 17 women). Adipocytes were isolated by collagenase (1 mg/ml) digestion. To determine cell size distribution and adipocyte number, aliquots of adipocytes were fixed with osmic acid and counted in a Coulter counter as previously described^19^. The study was approved by the ethics committee of the University of Leipzig (approval number: 159-12-21052012) and all subjects gave written informed consent. All methods were carried out in accordance with the approved guidelines.

### Analysis of human *HIF3A* mRNA expression

Briefly, human *HIF3A* mRNA expression was measured by qRT-PCR using TaqMan Gene Expression Assay (Applied Biosystems, Darmstadt, Germany). Total RNA was isolated from adipose tissue samples using the Qiacube System (Qiagen, Hilden, Germany), and 2 μg RNA were reverse transcribed with standard reagents (Life Technologies). Further details including PCR conditions are provided in the Supplemental material. The following Gene Expression Assay was used: Hs00541709_M1 (tagging the transcripts NM_022462.4, NM_152794.3, NM_152795.3, NM_152796.4). *HIF3A* mRNA expression was calculated relative to the mRNA expression of *hypoxanthine guanine phosphoribosyltransferase 1 (HPRT1)*, determined by the assay Hs01003267_M1 (Applied Biosystems, Darmstadt, Germany). Expression of *HIF3A* and *HPRT1* mRNA were quantified by using the second derivative maximum method of the TaqMan Software (Applied Biosystems).

For expression analysis of *HIF3A* in adipocytes and stromal vascular fraction, total RNA was isolated from adipocytes and stromal vascular fraction extracted from 35 paired samples of subcutaneous and visceral adipose tissue. 300 ng RNA were reverse transcribed with standard reagents and from each RT-PCR, 23.5 μl was amplified in a 40 μl PCR using the Taqman Gene Expression Assay and the TaqMan Fast Advanced Mastermix according to the manufacturer’s instruction. *HIF3A* mRNA expression was calculated relative to the mRNA expression of *HPRT1* mRNA or *18S rRNA*, determined by the assay Hs01003267_m1 (Applied Biosystems, Darmstadt, Germany).

### DNA extraction and bisulfite conversion

Briefly, genomic DNA was extracted using the DNeasy Blood and Tissue Kit (Qiagen, Hilden, Germany) and bisulfite conversion was performed using the Epitect Bisulfite Kit (Qiagen, Hilden, Germany) according to the manufacturer’s protocol.

### Determining CpG methylation levels

PCR and sequencing primers were designed using the PyroMark Assay Design 2.0 software (Qiagen, Hilden, Germany). DNA fragments were amplified from bisulfite-converted DNA using forward primer 5′-TGGTTGAAGGGTTATTTAGGG-3′and biotinylated reverse primer 5′-ACTCTATCCCACCCCTTTT-3′. The PCR reaction mixture and cycler program are provided in the Supplementary material. Bisulfite pyrosequencing was performed on a PyroMark Q96MD pyrosequencing system (Qiagen) using the PyroMark Gold Q96 CDT reagent kit (Qiagen) and the Pyro Q-CpG software (Qiagen). Percentage of methylation at eleven individual CpG sites within intron 1 of *HIF3A* were determined using three different sequencing primers (Assay 1: 5′-TTTAGGGGGTGTAGG-3′; Assay 2: 5′-GGTGAGATGATTTTATAGGAA-3′; Assay 3: 5′-GTTAAGAGGGGTTTTTATT-3′). Assay 1 included seven CpGs, Assay 2 only one CpG and Assay 3 three CpGs. The sixth CpG site in Assay 1, the CpG site in Assay 2 and the third CpG site in Assay 3 correspond to the CpG sites on the 450 K array reported elsewhere^11^. In our experience, the average methylation difference between technical replicates is approximately one percentage point.

### Genotyping of *HIF3A* SNPs

Genomic DNA was extracted from blood using the Quick Gene DNA whole blood Kit (Kurabo, Japan). Genotyping of the two previously reported SNPs rs8102595 (A/G) and rs3826795 (G/A)^11^ was performed using the TaqMan SNP Genotyping assay (Applied Biosystems; C _29247492_10; C_31640839_10). To assess genotyping reproducibility, a random ~5% selection of the sample were re-genotyped for all SNPs; all genotypes matched initial designated genotypes. Potential functional significance of the studied genetic variants was checked using the Regulome Database, which includes public datasets from GEO, the ENCODE project, and published literature^20^.

### Statistical Analyses

All non-normally distributed parameters were logarithmically transformed to approximate a normal distribution. To analyze differences in *HIF3A* methylation/expression levels between visceral and subcutaneous adipose tissue, paired two-tailed t-tests were applied. To test for group differences (e.g. lean vs. obese, NGT vs. T2D) two tailed *t-tests* were used. Pearson’s correlation coefficients were used to assess bivariate correlation with phenotypes related to obesity, fat distribution and glucose and insulin homeostasis. Linear regression models were used to control for confounders such as age, gender and BMI. To test SNPs for genetic associations with mRNA expression, DNA methylation and metabolic traits, linear regression analysis adjusted for respective covariates was applied. Association studies on type 2 diabetes (T2D) and obesity (lean with BMI < 25 kg/m^2^ vs. obese with BMI ≥ 30 kg/m^2^) were done using logistic regression analyses. P-values ≤ 0.05 were considered to provide nominal evidence for association. Two-sided p-values are reported without adjustments for multiple testing. The analysis of associations with quantitative traits was restricted to nondiabetic subjects to avoid diabetes status or treatment masking potential effects of the variants on these parameters. Statistical analyses were performed using SPSS statistics version 20.0.1 (SPSS, Inc., Chicago, IL, USA).

## Results

### *HIF3A* mRNA expression is fat depot related

**Figure 1:**
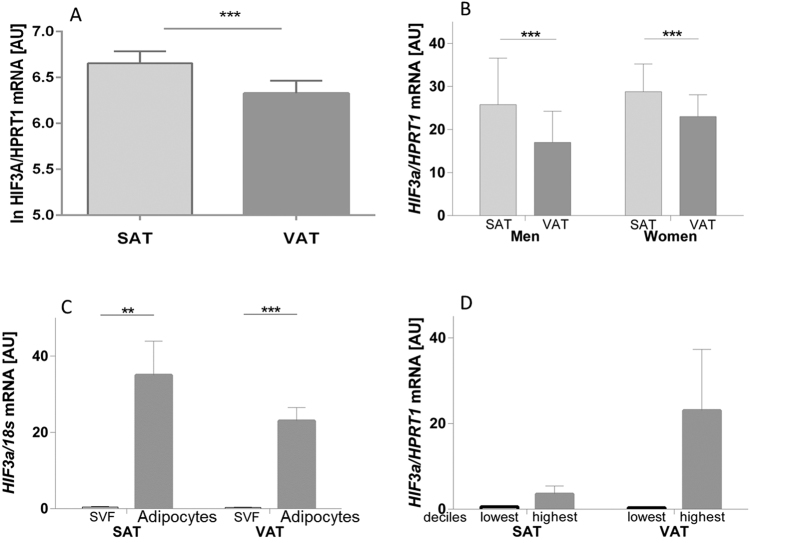
*HIF3A* mRNA expression in human subcutaneous (SAT, n = 584) and visceral (VAT, n = 588) adipose tissue. In the entire study cohort (**A**), but also in subgroups of men (SAT, n = 108; VAT, n = 110) and women (SAT, n = 231; VAT, n = 230) (Subjects with T2D were excluded from analysis) (**B**). Expression of *HIF3A* is significantly higher in subcutaneous (SAT) compared to visceral (VAT) adipose tissue. **(C)** *HIF3A* mRNA expression in adipocytes (n = 35) and cells of the stromal vascular fraction (SVF). *HIF3A* is significantly higher expressed in adipocytes compared to cells of the SVF in both compartments **(D)** *HIF3A* mRNA expression in relation to adipocyte cell size in subcutaneous (SAT) and visceral (VAT) adipose tissue. Individuals were categorized by mean SAT and VAT adipocyte size into deciles. Comparison of *HIF3A* mRNA expression between individuals with the lowest versus highest mean adipocyte size decile reveals that *HIF3A* is more highly expressed in subjects with higher mean adipocyte volume. Data are presented as means ± SEM. **p < 0.01, ***p < 0.001, AU-arbitrary units.

Analysis of paired subcutaneous and visceral adipose tissue samples revealed significantly higher *HIF3A* mRNA expression in subcutaneous compared to visceral adipose tissue (Fig. 1A). The fat depot differences in *HIF3A* expression could be confirmed in both genders (Fig. 1B). There was no significant difference in both subcutaneous and visceral adipose tissue *HIF3A* mRNA expression between individuals with normal glucose tolerance (NGT) and with type 2 diabetes (Supplementary Figure).

We further analyzed the contribution of adipocytes and stromal vascular fraction cells on whole adipose tissue *HIF3A* mRNA expression. Analysis of visceral and subcutaneous stromal vascular fraction showed significantly higher *HIF3A* mRNA levels in subcutaneous compared to visceral stromal vascular fraction (p < 0.05) (subcutaneous 0.56 ± 0.84 and visceral 0.37 ± 0.57). In paired samples of adipocytes and stromal vascular fraction cells we found significantly higher *HIF3A* expression in adipocytes compared to stromal vascular fraction cells both in subcutaneous and visceral fat compartments (Fig. 1C). There was no significant fat depot-related difference in *HIF3A* mRNA expression of isolated adipocytes. We further sought to determine *HIF3A* mRNA expression in relation to adipocyte cell size. Comparison of *HIF3A* mRNA expression between individuals with the lowest versus highest mean adipocyte size decile reveals that *HIF3A* is more highly expressed in subjects with higher mean adipocyte volume (Fig. 1D).

### *HIF3A* mRNA expression in adipose tissue correlates with parameters of obesity, systemic inflammation, glucose metabolism and mRNA expression of genes regulating adipogenesis (*leptin*, *PPARG*)

**Figure 2:**
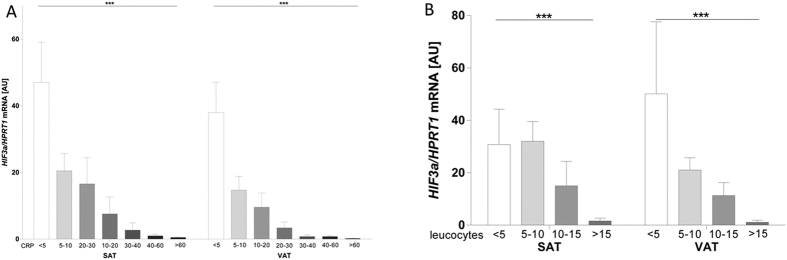
*HIF3A* mRNA expression in subcutaneous and visceral adipose tissue in relation to CRP serum concentration categories (n = 318) and leucocyte counts (n = 326). A significant inverse relationship between both CRP level (**A**) and leucocyte count (**B**) and expression of *HIF3A* in both compartments can be observed. Data are presented as means ± SEM. ***p < 0.001, AU-arbitrary units.

*HIF3A* mRNA expression in visceral and subcutaneous adipose tissue correlated significantly with BMI, body weight, waist and hip circumferences, abdominal visceral and subcutaneous fat area, %body fat, free fatty acid, triglyceride, alanine aminotransferase (ALAT), leptin serum concentrations and with the mRNA expression of *Leptin* (Table 2 and Supplementary Table 1). Furthermore, there were significant inverse correlations between subcutaneous and visceral adipose tissue *HIF3A* expression and age, CT ratio, adiponectin and C-reactive protein (CRP) serum concentrations (Fig. 2A) and leucocyte count (Fig. 2B). Only in visceral adipose tissue, *HIF3A* expression correlated with fasting plasma insulin, thyroid-stimulating hormone (TSH), high density lipoprotein (HDL)-cholesterol, gamma glutamyltransferase and the mRNA expression with PPARG (p < 0.05; Table 2).

After adjusting for age and gender, correlations between visceral and subcutaneous adipose tissue *HIF3A* mRNA expression and BMI, but also between visceral *HIF3A* mRNA expression and %body fat remained significant (Table 2). Correlations between subcutaneous and visceral adipose tissue *HIF3A* mRNA expression and waist, WHR, CRP level, leucocyte count and *leptin* mRNA expression remained significant after adjusting for age, gender and BMI (Table 2). After adjusting for covariates, free fatty acids only correlated with subcutaneous *HIF3A* mRNA expression and visceral *HIF3A* mRNA levels correlated with visceral mRNA expression of PPARG. In both fat depots, we found decreased *HIF3A* mRNA expression with increasing subcategories of both CRP serum concentrations and leucocyte counts (Fig. 2). To avoid a potential bias of systemic inflammation on *HIF3A* expression and its associations with anthropometric and metabolic traits, we performed correlation analyses only in individuals with CRP < 5mg/dl; however, the data remained unchanged (data not shown).

### Association of rs8102595 and rs3826795 with *HIF3A* DNA methylation, mRNA expression and metabolic traits

In the present study, we included 2 SNPs (rs8102595 and rs3826795) which have previously been shown to be associated with DNA methylation in a large cohort including >2000^11^. Both studied polymorphisms were in Hardy-Weinberg Equilibrium (p > 0.05) with following minor allele frequencies: rs8102595-10.8%, rs3826795-21.6%. There was no significant association between the SNPs and *HIF3A* mRNA expression in any of the two adipose tissue depots (Table 3). However, rs8102595 was nominally associated with *HIF3A* DNA methylation in visceral and subcutaneous adipose tissue (p < 0.05 after adjusting for age, gender and BMI; Table 3). Subjects carrying the minor allele (G) had a higher *HIF3A* DNA methylation in visceral adipose tissue, which was in line with the lower *HIF3A* mRNA expression in visceral adipose tissue (albeit not significant). Association analyses with parameters of obesity and fat distribution revealed a nominal association between rs3826795 and total cholesterol and the mean fat cell size of visceral adipose tissue (Supplementary Table 2). Rs8102595 showed an association with HDL-cholesterol, glucose infiltration rate and maximum fat cell size of subcutaneous adipose tissue (Table 3 and Supplementary Table 2).

### *HIF3A* DNA methylation in blood, subcutaneous and visceral adipose tissue

**Figure 3:**
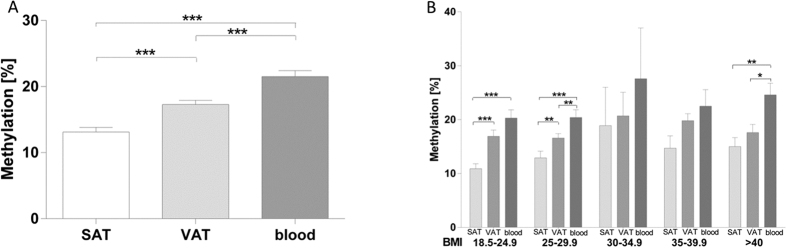
Methylation of cg22891070 in *HIF3A* in different tissues and in relation to BMI (n = 87). The *HIF3A* locus is significantly higher methylated in blood compared to SAT and VAT **(A)** The difference in methylation levels can be observed for all BMI groups **(B)** Methylation levels are higher in subjects with increased BMI **(B)**. Data are presented as means ± SEM. *p < 0.05, **p < 0.01, ***p < 0.001.

Methylation measured at the CpG site in Assay 2, corresponds to the published cg22891070, which has been reported to show the strongest correlation to BMI^11^. In our study, *HIF3A* DNA methylation at cg22891070 was significantly higher in blood (20.84 ± 7.74%) compared to subcutaneous (12.83 ± 6.82%; p < 0.001) and visceral adipose tissue (17.28 ± 5.61%; p < 0.001), whereas *HIF3A* DNA methylation in visceral adipose tissue was significantly higher than in subcutaneous adipose tissue (p <  0.001; Fig. 3A). In addition, DNA methylation at cg22891070 in visceral adipose tissue correlated significantly with hip (p < 0.01, r = 0.614), subcutaneous (p < 0.01, r = 0.651) and visceral fat mass (p < 0.05, r = 0.468) and inversely with the CT-ratio (p < 0.01, r = −0.653). Correlations between methylation in visceral adipose tissue and subcutaneous fat mass (p < 0.01), CT ratio (p < 0.01), hip (p < 0.01) and adiponectin (p < 0.05, r = −0.187) remained significant even after adjusting for age, gender and BMI. Furthermore, methylation of cg22891070 in subcutaneous adipose tissue correlated with CT ratio (p < 0.05, r = −0.571) and age (p < 0.05, r = −0.268). After adjusting for gender and BMI the correlation remained significantly for age (p = 0.032). Albeit not significant, in all analyzed tissues, obese individuals displayed a higher methylation of cg22891070 compared to lean and overweight individuals (Fig. 3B). The analyses including other tested CpG sites did not reveal correlations beyond those observed for cg22891070 (data not shown).

## Discussion

Recent studies revealed an association between BMI and methylation of *HIF3A* in whole blood and in adipose tissue^11^,^12^,^13^. It has been proposed that the HIF-system could play a role in mechanisms involved in the pathophysiology of adipose tissue-inflammation, obesity-induced insulin resistance and the etiology of obesity related diseases. We therefore sought to further elucidate the relationship between *HIF3A* mRNA expression in visceral and subcutaneous adipose tissue and obesity, but also methylation of CpG-sites in *HIF3A*. In summary, we show that *HIF3A* gene expression and methylation in adipose tissue are fat depot specific, and related to obesity and adipose tissue dysfunction.

We investigated the methylation and expression of *HIF3A* in two distinct fat depots, subcutaneous and visceral adipose tissue. We show that higher *HIF3A* mRNA expression in both subcutaneous and visceral adipose tissue is associated with higher BMI and obesity related traits. HIF3A has been shown to accelerate 3T3-L1 adipocyte differentiation and to induce the expression of adipocyte related genes^9^. Interestingly, we found higher adipose tissue *HIF3A* mRNA expression in individuals of the highest decile of mean adipocyte size (for both depots) compared to the lowest decile. This may suggest that *HIF3A* is involved in the determination of adipocyte size and may thereby contribute to adipose tissue expandability. Our results further support the hypothesis that expression of *HIF3A* might be induced in states of metabolic excess and mediate mechanisms involved in adipogenesis. Moreover, based on our data, the expression of *HIF3A* seems to be more pronounced in adipocytes compared to the stromal vascular fraction independent of the fat depot. To this end, adipocytes isolated from subcutaneous adipose tissue displayed higher expression levels of *HIF3A* than those isolated from visceral adipose tissue. Thus, the major proportion of *HIF3A* expression in adipose tissue might be attributed to primary adipocytes, which further supports the proposed regulatory role of HIF3A in adipogenesis. In further support of this, we found a positive correlation between the mRNA expression of *HIF3A* and *leptin* (in both visceral and subcutaneous adipose tissue) as well as *PPARG* (in visceral adipose tissue), two genes involved in the regulation of adipogenesis.

It is noteworthy, that *HIF3A* expression inversely correlated with CRP level and leucocyte count, suggesting down-regulation of the *HIF3A* expression in inflammatory states. Chronic inflammation in adipose tissue, liver and skeletal muscle are commonly associated with obesity^2^, which results in secondary pathologies like insulin resistance, hyperinsulinemia and glucose intolerance^3^,^21^. Obesity promoted relative hypoxia in adipocytes stimulates HIF1A-induction^5^,^6^, which then triggers the inflammation process by mediating the production of adipocyte-derived chemokines and adipose tissue macrophage accumulation^4^,^7^. HIF3A can inhibit HIF1A mediated signaling under certain circumstances^22^. The observed reduced expression of *HIF3A* in inflammatory states may facilitate increased HIF1A signaling, which in turn could activate an inflammatory cascade within adipose tissue.

*HIF3A* mRNA expression is regulated at different levels. Transcription of *HIF3A* can be induced by HIF1 via hypoxia response elements (HREs) in the promoter region and protein stability of HIF3α can be regulated in dependency of oxygen supply via the oxygen-dependent degradation domain (ODD)^22^,^23^,^24^. *HIF3A* expression has further been shown to be regulated by micro RNA (miRNA), thus to be modified on a post-transcriptional level^25^. These different mechanisms can supplement one another in fine tuning of *HIF3A* expression. We hypothesize that the complex regulation of *HIF3A* expression can be influenced by DNA methylation in various ways by interfering with different mechanisms of regulation. An association between BMI and methylation at three CpG-sites in intron 1 of *HIF3A* in whole blood and in adipose tissue has recently been identified by employing genome-wide DNA-methylation analyses^11^,^12^,^13^. In contrast, we did not find a correlation between BMI and *HIF3A* methylation. This may be due to the smaller sample size and a different composition of our cohort, which is characterized by a relatively high BMI (32.9 kg/m^2^), and thus, strongly differing from the previously reported cohorts with average BMI ranging between 24.2 and 28.3 kg/m^2^. Rönn *et al*. were able to replicate the association between methylation of *HIF3A* and BMI in a female cohort only^12^ and Demerath *et al*. showed *HIF3A* methylation to be associated with BMI only in one of three cohorts investigated^13^. Considering multiple isoforms of HIF3α^8^, it is plausible that methylation might be transcript-specific; yet, one would expect to observe consistent results upon expression analysis of the same transcript.

It is of note that the CpG sites at the *HIF3A* locus that were associated with BMI are situated within regions of open chromatin, suggesting that these sites lie in a regulatory region^11^,^26^. However, this regulation appears more complex than being dependent on methylation only. It is plausible that methylation of *HIF3A* results in altered expression profiles, networking with mechanisms in different stages of regulation. Yet, a linear effect between methylation and expression even of the same transcript cannot be confirmed.

Methylation analysis of *HIF3A* in our cohort revealed significant differences between methylation in blood, subcutaneous and visceral adipose tissue, being strongest in blood and weakest in subcutaneous adipose tissue. Since *HIF3A* mRNA expression in subcutaneous adipose tissue is higher than in visceral adipose tissue, it is possible that methylation could together with other regulatory mechanisms, cause a decrease in the expression of *HIF3A*. In line with this, rs8102595 was nominally associated with DNA methylation at cg22891070 in subcutaneous and visceral adipose tissue; thus supporting data by Dick *et al*.^11^ reporting associations of 2 SNPs (rs8102595 and rs3826795) with DNA methylation. Based on the Regulome Database^20^, rs3826795 might affect the binding of transcription factors POLR2A and SIN3A, and rs8102595 might influence DNA-protein binding. However, considering the lack of associations of the two SNPs with BMI, changes in *HIF3A* methylation seem to be mediated by obesity rather than promoting obesity itself^11^. It is also of note, that we did not observe an association between the SNPs and *HIF3A* mRNA expression in any of the two adipose tissue depots. We have to point out however, that the availability of the biomaterial (adipose tissue and blood samples) only allowed including 548 subjects for genotyping and for subsequent genotype-expression association analyses, which may have resulted in the lack of statistical power for correlation analyses.

In contrast to previous studies mostly investigating subcutaneous adipose tissue, the present study reveals mRNA expression and DNA methylation differences between subcutaneous and visceral adipose tissue. The two depots consist of different histological and biochemical compounds. The depot-specific expression of *HIF3A* may be important for the different functioning of the different depots. Whereas visceral adipose tissue is more vascular, innervated and contains a higher number of inflammatory and immune cells, subcutaneous adipose tissue has a higher preadipocyte differentiating capacity and a lower percentage of large adipocytes^27^. As *HIF3A* mRNA expression is higher in subcutaneous adipose tissue, possibly due to differences in methylation, this contributes to our assumption that HIF3α might be involved in preadipocyte differentiation, and that this process may be regulated by methylation, along with other factors. It is noteworthy that recently, we observed diminished hydroxymethylation levels in subcutaneous adipose tissue, as a measure of potential de-methylation mechanisms, which might be related to the higher number of pre-adipocytes in subcutaneous adipose tissue^28^.

We found methylation of *HIF3A* in both compartments to be correlated inversely with fat distribution, and methylation in VAT correlated significantly with subcutaneous fat mass. This suggests that methylation occurs rather in subjects with a preponderance of subcutaneous fat. We also detected an inverse association between age and methylation in subcutaneous adipose tissue, which leads to the assumption that the modification is dynamic and changes during lifetime.

Finally, it has to be acknowledged that the CpG site cg22891070 presented in our study is located between the 2 previously reported CpG islands^11^. Various *HIF3A* transcripts with different functions have been reported^22^ and it is also likely that they can be specifically affected by the methylation. Since the expression assay used in the present study tagged all potential *HIF3A* transcripts, we were not able to link cg22891070 to a specific transcript. However, in our own datasets based on genome-wide expression arrays (unpublished data) transcript variants 2 (NM_022462.4) and 3 (NM_152795.3) seem to be predominantly expressed in adipose tissue. Since Pasanen *et al*.^22^ suggested no functional relevance of the variant 3, it remains to be determined whether transcript variant 2 appears functionally relevant in adipose tissue.

In conclusion, our data suggest that *HIF3A* expression and methylation in adipose tissue is related to its dysfunction, making HIF3A an important factor involved in the complex etiology of obesity and associated comorbidities. HIF3A might function as an accelerator of adipogenesis in situations of excess of energetic supply and might contribute to the etiology of secondary obesity-induced pathologies by allowing a stronger induction of HIF1α-mediated proinflammatory signaling.

## Additional Information

**How to cite this article**: Pfeiffer, S. *et al*. *Hypoxia-inducible factor 3A* gene expression and methylation in adipose tissue is related to adipose tissue dysfunction. *Sci. Rep.* **6**, 27969; doi: 10.1038/srep27969 (2016).

## Supplementary Material

Supplementary Information

## Figures and Tables

**Table 1:** Anthropometric and metabolic characteristics of study participants.

	Total	Lean	Overweight	Obese	NGT	T2D	CRP < 5
N	865	88	73	704	484	343	347
Men/Women	288/577	40/48	36/37	212/492	155/329	124/219	129/218
Age (years)	49 ± 13	62 ± 15aaa	62 ± 14	46 ± 11ccc	47 ± 15ddd	51 ± 10	49 ± 13
BMI (kg/m2)	44.3 ± 12.6	22.1 ± 2.3aaa	27.2 ± 1.3bbb	48.9 ± 9.0ccc	40.9 ± 12.9ddd	49.4 ± 10.8	41.8 ± 11.7eee
Body weight (kg)	129 ± 39	63 ± 9aaa	78 ± 9bbb	142 ± 30ccc	119 ± 41ddd	143 ± 33	122 ± 38eee
Height (m)	1.6 ± 0.1	1.68 ± 0.09	1.69 ± 0.09	1.69 ± 0.09	1.69 ± 0.1	1.69 ± 0.1	1.70 ± 0.09
Waist (cm)	126.8 ± 26.2	77.0 ± 13.7aaa	96.8 ± 13.8bbb	137.1 ± 19.7ccc	116.2 ± 28.2ddd	142.6 ± 22.0	124.0 ± 27.9
Hip (cm)	129.1 ± 28.7	86.3 ± 9.7aaa	102.4 ± 11.4bbb	141.5 ± 21.0ccc	126.0 ± 30.3^dd^	135.7 ± 24.7	127.0 ± 28.5
WHR	0.95 ± 0.13	0.90 ± 0.11^aa^	0.94 ± 0.08	0.97 ± 0.14	0.91 ± 0.12ddd	1.05 ± 0.08	0.95 ± 0.13
Visceral Fat area (cm^2^)	240 ± 172	45 ± 29aaa	119 ± 60bbb	313 ± 154ccc	177 ± 142ddd	392 ± 150	209 ± 175
SC fat area (cm^2^)	1093 ± 789	52 ± 26aaa	273 ± 171bbb	1509 ± 559ccc	992 ± 817ddd	1386 ± 671	920 ± 814^e^
CT ratio (vis/sc)	0.4 ± 0.5	1.9 ± 0.9aaa	0.5 ± 0.3^bb^	0.2 ± 0.1ccc	0.3 ± 0.4	0.5 ± 0.7	0.4 ± 0.5
Body Fat (%)	41.4 ± 11.5	19.0 ± 3.5	24.3 ± 3.9	45.1 ± 8.0	38.5 ± 13.0	44.3 ± 9.4	39.1 ± 11.0
CRP (mg/dl)	11.4 ± 14.4	14.9 ± 22.9	9.1 ± 13.1	11.2 ± 13.1	11.2 ± 15.6	12.1 ± 13.2	2.4 ± 1.5
IL-6 (pg/ml)	6.0 ± 5.2	2.2 ± 3.3	2.8 ± 2.4	7.0 ± 5.3	5.2 ± 4.6	7.5 ± 6.0	4.1 ± 1.3
HbA1c (%)	6.1 ± 1.2	5.3 ± 0.4	5.7 ± 0.6	6.1 ± 1.2	5.5 ± 0.5	6.9 ± 1.4	6.0 ± 1.0
oGTT2h (mmol/l)	7.0 ± 2.6	6.0 ± 1.0	6.1 ± 0.9	7.4 ± 2.9	6.3 ± 1.0	14.8 ± 5.9	6.6 ± 1.7
FPG (mmol/l)	6.5 ± 2.5	5.5 ± 1.0	5.9 ± 1.5	6.7 ± 2.7	5.4 ± 1.0	8.1 ± 3.2	6.1 ± 2.1
FPI (pmol/)	123.1 ± 133.8	10.8 ± 20.6	68.0 ± 92.5	146.4 ± 137.2	62.4 ± 70.8	206.2 ± 156.4	109.7 ± 121.7
GIR (μmol/kg/min)	75.1 ± 33.4	102.5 ± 18.5	77.7 ± 25.8	56.9 ± 31.2	90.6 ± 21.4	30.5 ± 23.5	85.3 ± 28.3
Total cholesterol (mmol/l)	4.9 ± 1.0	5.1 ± 0.8	5.0 ± 1.1	4.9 ± 1.0	4.9 ± 1.0	4.9 ± 1.0	4.9 ± 1.0
HDL-C (mmol/l)	1.2 ± 0.3	1.7 ± 0.5	1.4 ± 0.3	1.1 ± 0.3	1.3 ± 0.4	1.1 ± 0.3	1.2 ± 0.4
LDL-C (mmol/l)	3.1 ± 0.9	2.8 ± 1.0	3.2 ± 0.8	3.1 ± 0.9	3.1 ± 0.9	3.0 ± 0.8	3.1 ± 0.9
FFA (mmol/l)	0.5 ± 0.4	0.2 ± 0.2	0.3 ± 0.3	0.6 ± 0.3	0.3 ± 0.3	0.8 ± 0.3	0.5 ± 0.3
TG (mmol/l)	1.8 ± 1.1	1.1 ± 0.4aaa	1.2 ± 0.5	1.9 ± 1.1ccc	1.4 ± 0.9ddd	2.1 ± 1.1	1.8 ± 1.1
Leptin (ng/ml)	39.3 ± 24.2	4.8 ± 3.7aaa	12.4 ± 7.0bbb	45.4 ± 21.8ccc	37.1 ± 23.9	41.2 ± 25.0	35.6 ± 22.7^e^
Adiponectin (μg/ml)	6.9 ± 4.4	14.3 ± 6.2aaa	8.8 ± 3.5bbb	6.0 ± 3.3ccc	8.5 ± 4.7ddd	4.9 ± 3.1	7.1 ± 4.2
Albumin (g/L)	28.1 ± 18.9	32.9 ± 7.7	34.4 ± 13.2	26.9 ± 20.2	27.1 ± 18.8	28.6 ± 19.6	29.5 ± 20.0
ALAT (μkat/l)	0.6 ± 0.5	0.4 ± 0.3aaa	0.5 ± 0.3	0.7 ± 0.5ccc	0.6 ± 0.4^d^	0.7 ± 0.5	0.7 ± 0.4
ASAT (μkat/l)	0.6 ± 2.3	0.4 ± 0.3	0.4 ± 0.2	0.6 ± 2.5	0.6 ± 3.0	0.6 ± 0.5	0.5 ± 0.3
gGT (μkat/l)	0.9 ± 1.3	1.0 ± 1.4	1.0 ± 1.5	0.8 ± 1.3	0.8 ± 1.0^d^	1.0 ± 1.7	0.7 ± 0.9^e^
TSH (mU/l)	1.9 ± 7.9	1.5 ± 2.1	1.5 ± 1.9	2.1 ± 8.8	1.7 ± 1.8	2.3 ± 12.4	1.4 ± 1.0
fT3 (pg/ml)	4.6 ± 0.9	4.5 ± 1.0	4.4 ± 0.7	4.7 ± 0.9^c^	4.5 ± 0.9	4.7 ± 0.9	4.6 ± 0.9
fT4 (pmol/l)	17.1 ± 1.4	17.2 ± 3.3	17.5 ± 3.2	16.9 ± 3.5	16.9 ± 3.4^d^	17.4 ± 3.4	17.6 ± 3.3^e^
Leucocytes/nl	8.1 ± 2.7	7.5 ± 3.2	7.4 ± 3.1	8.2 ± 2.6^c^	2.1 ± 0.5	2.1 ± 0.5	7.4 ± 2.2^e^
Erythrocytes (Mio/μl)	4.7 ± 0.8	4.6 ± 2.7	4.3 ± 0.9	4.7 ± 0.4	4.7 ± 1.0	4.7 ± 0.4	4.7 ± 0.4
Thrombocytes (10^9^/l)	260 ± 81	252 ± 108	241 ± 71	261 ± 79	261 ± 73	259 ± 88	232 ± 66^e^

Data are means ± SD; ^a,b,c,d,e^p < 0.05, ^aa,bb,cc,dd,ee^p < 0.01, ^aaa,bbb,ccc,ddd,eee^p < 0.001 for comparison between (a) lean and obese, (b) lean and overweight, (c) overweight and obese, (d) type 2 diabetes subjects (T2D) and subjects with normal glucose tolerance (NGT) and (e) CRP < 5 and the entire cohort. 51 subjects with type 1 diabetes or impaired glucose tolerance were not considered for group comparison. BMI – Body Mass Index, WHR – waist-to-hip ratio, sc - subcutaneous, TG – Triglycerides, ALAT – alanine aminotransferase, ASAT - aspartate aminotransferase, gGT - Gamma-glutamyl transferase, TSH – thyroid-stimulating hormone, fT3 – free triiodothyronine, fT4 – free tetraiodothyronine, CRP – C-reactive protein, IL-6 – Interleukin 6, HbA1c – Glycohemoglobin, oGTT – oral Glucose Tolerance Test, FPG – Fasting plasma glucose, FPI – Fasting plasma insulin, GIR – Glucose infusion rate during the steady state of an euglycemic hyperinsulinemic clamp, HDL-C – high Density Lipoprotein Cholesterol, LDL-C – Low Density Lipoprotein Cholesterol, FFA – Free Fatty Acids.

**Table 2:** Correlation analyses of subcutaneous and visceral adipose tissue *HIF3A* mRNA expression with metabolic parameters, methylation levels and mRNA expression of *leptin* and *PPARG*.

	*HIF3A*mRNA Expression in subcutaneous adipose tissue			*HIF3A* mRNA Expression in visceral adipose tissue		
	r	p-value	adj. p-value	r	p-value	adj. p-value
Age (years)	−0.23	4.61 × 10^−5^	**0.032**	−0.237	3.08 × 10^−5^	0.076
BMI (kg/m^2^)	0.239	2.86 × 10^−5^	**0.017**^a^	0.283	5.46 × 10^−7^	**8.84** × **10**^−**4**a^
Body weight (kg)	0.235	5.56 × 10^−5^	0.467^a^	0.263	5.45 × 10^−6^	0.280^a^
Height (m)	0.044	0.458	0.467	0.001	0.983	0.538
Waist (cm)	0.472	8.41 × 10^−9^	**0.010**	0.515	1.89 × 10^−10^	**0.048**
Hip (cm)	0.387	2.13 × 10^−5^	0.425	0.442	6.73 × 10^−7^	0.628
WHR	0.172	0.067	**0.018**	0.139	0.135	**0.033**
Visceral fat area (cm^2^)	0.391	3.19 × 10^−5^	0.636	0.442	1.71 × 10^−6^	0.479
SC fat area (cm^2^)	0.392	2.99 × 10^−5^	0.240	0.465	4.06 × 10^−7^	0.604
CT ratio (sc/vis)	−0.259	7.04 × 10^−3^	0.165	−0.319	7.80 × 10^−4^	0.325
Body fat (%)	0.324	0.017	0.055^a^	0.442	8.23 × 10^−4^	**0.013**^a^
CRP (mg/dl)	−0.138	0.021	**1.8** × **10**^−**3**^	−0.153	0.010	**3.19** × **10**^−**4**^
Leucocytes/nl	−0.127	0.032	**3.05** × **10**^−**3**^	−0.133	0.024	**1.13** × **10**^−**3**^
Met Blood (%)	0.054	0.720	0.618	0.023	0.876	0.772
Met SAT (%)	−0.054	0.687	0.482	−0.088	0.498	0.345
Met VAT (%)	0.060	0.648	0.667	−0.045	0.729	0.757
Leptin mRNA sc	0.227	2.82 × 10^−4^	**2.37** × **10**^−**4**^	0.216	5.28 × 10^−4^	**1.41** × **10**^−**3**^
Leptin mRNA vis	0.117	0.063	**0.043**	0.195	2.0 × 10^−3^	**2.60** × **10**^−**3**^
PPARG mRNA sc	0.001	0.977	0.939	0.002	0.967	0.878
PPARG mRNA vis	0.050	0.253	0.439	0.111	0.010	**0.023**

r - correlation coefficient (Pearson adj. – p-value adjusted to age, sex and BMI), ^a^adjusted for sex and age; BMI – Body Mass Index, WHR – waist-to-hip ratio, sc - subcutaneous, CRP – C-reactive protein, Met Blood (%)/Met SAT (%)/Met VAT (%) - Methylation of cg22891070 in *HIF3A* in blood/SAT/VAT.

**Table 3:** Association of rs8102595 and rs3826795 with anthropometric and metabolic characteristics, mRNA expression and DNA methylation.

	rs8102595			rs3826795		
	A/A	A/G + G/G	p-value	A/A+ A/G	G/G	p-value
N	446	95		208	336	
Men/Women	151/295	32/63		73/135	110/226	
Age	52.83 ± 15.79	55.48 ± 15.44	0.482	49.56 ± 15.31	50.72 ± 14.69	0.278
BMI (kg/m^2^)	43.48 ± 13.74	42.51 ± 13.50	0.239	43.64 ± 14.04	42.93 ± 13.32	0.908
Body weight (kg)	126.86 ± 42.81	124.57 ± 40.14	0.680	128.42 ± 45.54	124.60 ± 41.15	0.769
Height (m)	1.69 ± 0.09	1.69 ± 0.9	0.628	1.69 ± 0.09	1.69 ± 0.09	0.763
Waist (cm)	124.26 ± 29.98	121.84 ± 30.09	0.798	124.46 ± 30.43	122.85 ± 29.87	0.935
Hip (cm)	130.53 ± 28.99	128.59 ± 28.38	0.851	129.54 ± 28.38	130.08 ± 29.56	0.676
WHR	0.95 ± 0.13	0.96 ± 0.16	0.316	0.96 ± 0.16	0.94 ± 0.12	0.921
VAT area (cm^2^)	242.93 ± 173.84	237.02 ± 159.92	0.575	256.05 ± 183.40	228.98 ± 159.97	0.674
SAT area (cm^2^)	1095.74 ± 795.48	1129.73 ± 819.78	0.536	1122.85 ± 774.80	1094.46 ± 817.64	0.902
VAT mean	123.00 ± 20.82	122.08 ± 20.60	0.999	119.69 ± 25.71	124.66 ± 17.25	**0.014**
SAT mean	127.37 ± 19.89	127.51 ± 17.42	0.486	126.50 ± 19.04	127.99 ± 19.84	0.334
VAT max	209.23 ± 58.51	230.21 ± 96.06	0.060	210.73 ± 74.84	213.66 ± 63.47	0.109
SAT max	214.28 ± 70.88	249.22 ± 110.69	**1.23 × 10**^**−3**^	224.71 ± 80.22	217.94 ± 79.94	0.987
CT ratio (vis/sc)	0.47 ± 0.63	0.38 ± 0.30	0.922	0.40 ± 0.42	0.48 ± 0.66	0.826
Body fat (%)	41.95 ± 11.35	42.26 ± 11.72	0.496	41.15 ± 11.88	42.57 ± 11.11	0.607
CRP (mg/dl)	12.04 ± 15.09	11.20 ± 16.05	0.935	13.09 ± 15.67	11.34 ± 15.49	0.198
Leucocytes/nl	8.21 ± 2.88	8.08 ± 2.50	0.743	8.42 ± 3.22	8.00 ± 2.48	0.155
Blood Met (%)	20.99 ± 8.07	22.31 ± 5.11	0.143	21.43 ± 7.36	21.27 ± 7.56	0.811
Met SAT (%)	11.95 ± 5.86	16.34 ± 6.54	**0.011**	13.56 ± 7.38	12.69 ± 5.83	0.784
Met VAT (%)	17.04 ± 5.61	19.69 ± 6.10	**0.038**	18.20 ± 4.41	17.46 ± 6.18	0.401
SAT *HIF3A* mRNA	21.08 ± 72.62	7.43 ± 40.53	0.209	11.58 ± 49.82	22.60 ± 76.47	0.660
VAT *HIF3A* mRNA	23.92 ± 106.19	10.45 ± 50.03	0.073	16.80 ± 82.25	24.09 ± 106.69	0.729

Due to the low minor allele frequency (MAF) of the studied polymorphisms, subjects homozygous for the minor alleles (n = 3 for rs8102595, n = 16 for rs3826795) were combined with heterozygous groups (i.e. dominant mode of inheritance was used for statistical analyses).

p-value adjusted for age, gender and BMI and diabetes status; BMI – Body Mass Index, WHR – waist-to-hip ratio, SAT – subcutaneous adipose tissue, VAT- visceral adipose tissue CRP – C-reactive protein, Met Blood (%)/Met SAT (%)/Met VAT (%) - Methylation of cg22891070 in *HIF3A* in blood/SAT/VAT, *HIF3A* mRNA – mRNA expression of *HIF3A* in subcutaneous/visceral adipose tissue.
