# Supplementary material for: Hypoxia-inducible factor 3A gene expression and methylation in adipose tissue is related to adipose tissue dysfunction
Source: Sci Rep. 2016 Jun 27;6:27969. doi: 10.1038/srep27969 (PMC4921806; doi:10.1038/srep27969)
Supplement: Supplementary Information [file srep27969-s1.doc]

***Hypoxia-inducible factor 3A* gene expression and methylation in adipose tissue is related to adipose tissue dysfunction**

Susanne Pfeiffer^1*^, Jacqueline Krüger^2*^, Anna Maierhofer^3^, Yvonne Böttcher^2^, Nora Klöting^1,2^, Nady El Hajj^3^, Dorit Schleinitz^2^, Michael R. Schön^4^, Arne Dietrich^2,5^, Mathias Fasshauer^1,2^, Tobias Lohmann^6^, Miriam Dreßler^6^, Michael Stumvoll^1^, Thomas Haaf^3^, Matthias Blüher^1^, Peter Kovacs^2^

^1^Department of Medicine, Dermatology und Neurology, Department of Endocrinology und Nephrology, University of Leipzig, Leipzig, Germany

^2^Leipzig University Medical Center, IFB AdiposityDiseases, University of Leipzig, Leipzig, Germany

^3^Institute of Human Genetics, University of Würzburg, Würzburg, Germany

^4^Clinic of Visceral Surgery, Städtisches Klinikum Karlsruhe, Karlsruhe, Germany

^5^Department of Surgery, University of Leipzig, Leipzig, Germany

^6^Municipal Clinic Dresden-Neustadt, Dresden, Germany

**Supplemental Material**

**Material and Methods**

**Analysis of human *HIF3A* mRNA expression**

Briefly, human *HIF3*α mRNA expression was measured by qRT-PCR using TaqMan Gene Expression Assay (Applied Biosystems, Darmstadt, Germany), and fluorescence was detected on a TaqMan Quant Studio 6 Flex Real-Time PCR-System (Applied Biosystems, Darmstadt, Germany). Total RNA was isolated from AT samples using the Qiacube System (Qiagen, Hilden, Germany), and 2 µg RNA were reverse transcribed with standard reagents (Life Technologies). From each RT-PCR**,** 2µl was amplified in a 20µl PCR using the Taqman Gene Expression Assay (Applied Biosystems, Darmstadt, Germany) and the TaqMan Fast Advanced Mastermix (Applied Biosystems, Darmstadt, Germany). Samples were incubated in the Quant Studio 6 Flex Real-Time PCR-System (Applied Biosystems, Darmstadt, Germany) for an initial denaturation at 95°C for 20s, followed by 45 PCR cycles, each cycle consisting of 95°C for 1s and 60°C for 20s. The following Gene Expression Assay was used: Hs00541709_M1 (tagging the transcripts NM_022462.4, NM_152794.3, NM_152795.3 and NM_152796.4). *HIF3A* mRNA expression was calculated relative to the mRNA expression of *HPRT1* mRNA, determined by a premixed assay on demand for *HPRT1* mRNA (Hs01003267_M1, Applied Biosystems, Darmstadt, Germany). Expression of *HIF3A* and *HPRT1* mRNA were quantified by using the second derivative maximum method of the TaqMan Software (Applied Biosystems).

For expression analysis of *HIF3A* in adipocytes and SVF, total RNA was isolated from adipocytes and SVF extracted from paired samples of SAT and VAT. 305 ng RNA were reverse transcribed with standard reagents and from each RT-PCR**,** 23.5µl was amplified in a 40µl PCR using the Taqman Gene Expression Assay and the TaqMan Fast Advanced Mastermix according to the manufacturer’s instruction. *HIF3A* mRNA expression was calculated relative to the mRNA expression of *hypoxanthine guanine phosphoribosyltransferase 1 (HPRT1)* mRNA or *18S rRNA* (for isolated adipocytes only), determined by a premixed assay on demand (Hs01003267_m1; Applied Biosystems, Darmstadt, Germany).

**DNA extraction and bisulfite conversion**

Genomic DNA was extracted using the DNeasy Blood and Tissue Kit (Qiagen, Hilden, Germany) and bisulfite conversion was performed using the Epitect Bisulfite Kit (Qiagen, Hilden, Germany) according to the manufacturer’s protocol. PCRs were carried out to amplify DNA fragments for pyrosequencing. The following primers were used (Metabion, Martiensried, Germany): Forward 5’-TGGTTGAAGGGTTATTTAGGG-3’; reverse carrying a biotin label at its 5’-end 5’-ACTCTATCCCACCCCTTTT-3’. The PCR reaction mixture for pyrosequencing consisted of 5 µl 10x PCR buffer with MgCl_2_ (Roche Diagnostics, Mannheim, Germany), 1 µl (10 mM dNTPs) PCR Grade Nucleotide Mix (Roche Diagnostics), 2.5 µl (10 pmol/µl) of forward and reverse primer (Metabion, München-Planegg, Germany), 0.4 µl (5 U/µl) FastStart Taq DNA Polymerase (Roche Diagnostics), 2 µl of bisulfite-converted DNA and 36.6 µl PCR-grade water. Amplifications were performed with an initial denaturation step at 95°C for 5 min, 38 cycles of 95°C for 30 s, 60°C for 30 s, and 72°C for 45 s, and a final extension step at 72°C for 5 min.

**Results**

**Supplementary Table 1. Correlation analyses between subcutaneous and visceral adipose tissue *HIF3A* mRNA expression and study parameters.**

|  | *HIF3A* mRNA Expression  in subcutaneous adipose tissue | | | *HIF3A* mRNA Expression  in visceral adipose tissue | | |
| --- | --- | --- | --- | --- | --- | --- |
|  | r | p-value | adj. p-value | r | p-value | adj. p-value |
| Age (years) | -0.23 | 4.61x10^-5^ | **0.032** | -0.237 | 3.08x10^-5^ | 0.076 |
| BMI (kg/m²) | 0.239 | 2.86x10^-5^ | **0.017**^a^ | 0.283 | 5.46x10^-7^ | **8.84x10^-4^**^a^ |
| Body weight (kg) | 0.235 | 5.56x10^-5^ | 0.467^a^ | 0.263 | 5.45x10^-6^ | 0.280^a^ |
| Height (m) | 0.044 | 0.458 | 0.467 | 0.001 | 0.983 | 0.538 |
| Waist (cm) | 0.472 | 8.41x10^-9^ | **0.010** | 0.515 | 1.89x10^-10^ | **0.048** |
| Hip (cm) | 0.387 | 2.13x10^-5^ | 0.425 | 0.442 | 6.73x10^-7^ | 0.628 |
| WHR | 0.172 | 0.067 | **0.018** | 0.139 | 0.135 | **0.033** |
| Visceral fat area (cm²) | 0.391 | 3.19x10^-5^ | 0.636 | 0.442 | 1.71x10^-6^ | 0.479 |
| SC fat area (cm²) | 0.392 | 2.99x10^-5^ | 0.240 | 0.465 | 4.06x10^-7^ | 0.604 |
| CT ratio (sc/vis) | -0.259 | 7.04x10^-3^ | 0.165 | -0.319 | 7.80x10^-4^ | 0.325 |
| Body fat (%) | 0.324 | 0.017 | 0.055^a^ | 0.442 | 8.23x10^-4^ | **0.013** |
| CRP (mg/dl) | -0.138 | 0.021 | **1.8x10^-3^** | -0.153 | 0.010 | **3.19x10^-4^** |
| FPG (mmol/l) | 0.077 | 0.200 | 0.180 | 0.017 | 0.770 | 0.784 |
| FPI (pmol/l) | 0.130 | 0.181 | 0.161 | 0.250 | 8.21x10^-3^ | 0.634 |
| Total Cholesterol (mmol/l) | -0.017 | 0.827 | 0.673 | -0.010 | 0.891 | 0.586 |
| HDL-C (mmol/l) | -0.138 | 0.141 | 0.913 | -0.211 | 0.022 | 0.602 |
| LDL-C | 0.109 | 0.249 | 0.896 | 0.111 | 0.236 | 0.802 |
| FFA (mmol/l) | 0.442 | 1.99x10^-5^ | **8.97x10^-3^** | 0.401 | 1.43x10^-4^ | 0.063 |
| TG (mmol/l) | 0.218 | 3.40x10^-3^ | 0.085 | 0.196 | 7.63x10^-3^ | 0.262 |
| Leptin (ng/ml) | 0.413 | 2.14x10^-5^ | 0.866 | 0.461 | 1.78x10^-6^ | 0.918 |
| Adiponectin (µg/ml) | -0.290 | 3.47x10^-3^ | 0.415 | -.0348 | 4.09x10^-4^ | 0.241 |
| Albumin (g/L) | -0.328 | 0.072 | 0.159 | -0.217 | 0.225 | **0.015** |
| ALAT (µkat/l) | 0.176 | 3.31x10^-3^ | 0.303 | 0.135 | 0.023 | 0.980 |
| gGT (µkat/l) | -0.101 | 0.095 | 0.226 | -0.148 | 0.014 | **0.040** |
| TSH (mU/l) | 0.091 | 0.153 | 0.850 | 0.128 | 0.043 | 0.471 |
| Leucocytes/nl | -0.127 | 0.032 | **3.05x10^-3^** | -0.133 | 0.024 | **1.13x10^-3^** |
| Met Blood (%) | 0.054 | 0.720 | 0.618 | 0.023 | 0.876 | 0.772 |
| Met SAT (%) | -0.054 | 0.687 | 0.482 | -0.088 | 0.498 | 0.345 |
| Met VAT (%) | 0.060 | 0.648 | 0.667 | -0.045 | 0.729 | 0.757 |

r - correlation coefficient (Pearson adj. – p-value adjusted to age, gender and BMI, ^a^ adjusted for gender and age; BMI – Body Mass Index, WHR – waist-to-hip ratio, sc - subcutaneous, CRP – C-reactive protein, FPI – Fasting plasma insulin, HDL-C – high Density Lipoprotein Cholesterol, FFA – Free Fatty Acids, TG – Triglycerides, ALAT – alanine aminotransferase, gGT -Gamma-glutamyl transferase, TSH – thyroid-stimulation hormone, Met Blood (%)/ Met SAT (%) / Met VAT (%) - Methylation of cg22891070 in *HIF3A* in blood / SAT/ VAT

**Supplementary Table 2. Association of rs8102595 and rs3826795 with anthropometric and metabolic characteristics, mRNA expression and DNA methylation.**

|  |  | rs8102595 |  |  | rs3826795 |  |
| --- | --- | --- | --- | --- | --- | --- |
|  | A/A | A/G + G/G | p-value | A/A+ A/G | G/G | p-value |
| N | 446 | 95 |  | 208 | 336 |  |
| Men/Women | 151/295 | 32/63 |  | 73/135 | 110/226 |  |
| Age | 52.83±15.79 | 55.48±15.44 | 0.482 | 49.56±15.31 | 50.72±14.69 | 0.278 |
| BMI (kg/m²) | 43.48±13.74 | 42.51±13.50 | 0.239 | 43.64±14.04 | 42.93±13.32 | 0.908 |
| Body weight (kg) | 126.86±42.81 | 124.57±40.14 | 0.680 | 128.42±45.54 | 124.60±41.15 | 0.769 |
| Height (m) | 1.69±0.09 | 1.69±0.9 | 0.628 | 1.69±0.09 | 1.69±0.09 | 0.763 |
| Waist (cm) | 124.26±29.98 | 121.84±30.09 | 0.798 | 124.46±30.43 | 122.85±29.87 | 0.935 |
| Hip (cm) | 130.53±28.99 | 128.59±28.38 | 0.851 | 129.54±28.38 | 130.08±29.56 | 0.676 |
| WHR | 0.95±0.13 | 0.96±0.16 | 0.316 | 0.96±0.16 | 0.94±0.12 | 0.921 |
| VAT area (cm²) | 242.93±173.84 | 237.02±159.92 | 0.575 | 256.05±183.40 | 228.98±159.97 | 0.674 |
| SAT area (cm²) | 1095.74±795.48 | 1129.73±819.78 | 0.536 | 1122.85±774.80 | 1094.46±817.64 | 0.902 |
| VAT mean | 123.00±20.82 | 122.08±20.60 | 0.999 | 119.69±25.71 | 124.66±17.25 | **0.014** |
| SAT mean | 127.37±19.89 | 127.51±17.42 | 0.486 | 126.50±19.04 | 127.99±19.84 | 0.334 |
| VAT max | 209.23±58.51 | 230.21±96.06 | 0.060 | 210.73±74.84 | 213.66±63.47 | 0.109 |
| SAT max | 214.28±70.88 | 249.22±110.69 | **1.23x10^-3^** | 224.71±80.22 | 217.94±79.94 | 0.987 |
| CT ratio (vis/sc) | 0.47±0.63 | 0.38±0.30 | 0.922 | 0.40±0.42 | 0.48±0.66 | 0.826 |
| Body fat (%) | 41.95±11.35 | 42.26±11.72 | 0.496 | 41.15±11.88 | 42.57±11.11 | 0.607 |
| CRP (mg/dl) | 12.04±15.09 | 11.20±16.05 | 0.935 | 13.09±15.67 | 11.34±15.49 | 0.198 |
| IL-6 (pg/ml) | 6.05±5.24 | 5.17±3.71 | 0.880 | 6.16±4.54 | 5.79±5.39 | 0.286 |
| HbA1C (%) | 6.07±1.11 | 5.82±0.86 | 0.443 | 6.00±1.10 | 6.02±1.05 | 0.766 |
| oGTT2h (mmol/l) | 6.99±2.41 | 7.49±3.97 | 0.663 | 7.57±3.40 | 6.74±2.22 | 0.064 |
| FPG (mmol/l) | 6.34±2.17 | 5.83±0.98 | 0.827 | 6.34±2.45 | 6.22±1.77 | 0.904 |
| FPI (pmol/) | 131.78±141.75 | 107.18±98.96 | 0.297 | 124.28±118.40 | 127.54±145.26 | 0.729 |
| GIR (µmol/kg/min) | 75.66±33.87 | 65.78±35.86 | **0.042** | 73.75±33.57 | 73.58±35.24 | 0.567 |
| Total cholesterol (mmol/l) | 4.87±0.99 | 4.72±097 | 0.130 | 4.99±1.06 | 4.75±0.93 | **0.019** |
| HDL-C (mmol/l) | 1.24±0.41 | 1.22±0.46 | **0.027** | 1.25±0.40 | 1.23±0.42 | 0.418 |
| LDL-C (mmol/l) | 3.11±0.94 | 3.02±1.12 | 0.143 | 3.19±1.02 | 3.01±0.93 | 0.167 |
| FFA (mmol/l) | 0.58±0.41 | 0.54±0.39 | 0.525 | 0.57±0.43 | 0.57±0.39 | 0.076 |
| TG (mmol/l) | 1.65±0.89 | 1.49±0.66 | 0.654 | 1.69±0.96 | 1.58±0.78 | 0.444 |
| Leptin (ng/ml) | 37.12±22.14 | 40.64±23.96 | 0.313 | 35.43±21.13 | 39.15±23.34 | 0.655 |
| Adiponectin (µg/ml) | 7.93±4.97 | 6.59±3.36 | 0.091 | 7.17±4.67 | 8.11±4.75 | 0.095 |
| Albumin (g/l) | 40-95±9.62 | 40.59±9.43 | 0.648 | 39.37±10.74 | 41.84±8.40 | 0.086 |
| ALAT (µkat/l) | 0.65±0.50 | 0.58±0.49 | 0.360 | 0.65±0.53 | 0.62±0.47 | 0.767 |
| ASAT (µkat/l) | 0.71±3.18 | 0.52±0.31 | 0.382 | 0.89±0.65 | 0.54±0.28 | 0.742 |
| gGT (µkat/l) | 0.89±1.65 | 0.81±0.77 | 0.679 | 0.85±1.33 | 0.88±1.64 | 0.960 |
| TSH (mU/l) | 2.317±10.87 | 1.735±2.64 | 0.296 | 2.96±15.74 | 1.73±2.06 | 0.071 |
| fT3 (pg/ml) | 4.66±0.99 | 4.71±0.81 | 0.348 | 4.76±1.03 | 4.62±0.92 | 0.609 |
| fT4 (pmol/l) | 17.15±3.47 | 17.81±3.63 | 0.354 | 16.78±2.68 | 17.65±3.71 | 0.222 |
| Blood Met (%) | 20.99±8.07 | 22.31±5.11 | 0.143 | 21.43±7.36 | 21.27±7.56 | 0.811 |
| Met SAT (%) | 11.95±5.86 | 16.34±6.54 | **0.011** | 13.56±7.38 | 12.69±5.83 | 0.784 |
| Met VAT (%) | 17.04±5.61 | 19.69±6.10 | **0.038** | 18.20±4.41 | 17.46±6.18 | 0.401 |
| SAT *HIF3α* mRNA | 21.08±72.62 | 7.43±40.53 | 0.209 | 11.58±49.82 | 22.60±76.47 | 0.660 |
| VAT *HIF3α* mRNA | 23.92±106.19 | 10.45±50.03 | 0.073 | 16.80±82.25 | 24.09±106.69 | 0.729 |
| Leucoytes/nl | 8.21±2.88 | 8.08±2.50 | 0.743 | 8.42±3.22 | 8.00±2.48 | 0.155 |
| Erythrocytes (Mio/µl) | 4.79±1.05 | 4.62±0.50 | 0.230 | 4.65±0.59 | 4.82±1.16 | 0.237 |
| Thrombocytes x10^9^/l | 257.94±82.94 | 291.52±101.69 | 0.089 | 261.59±75.91 | 263.98±93.83 | 0.762 |

Due to the low minor allele frequency (MAF) of the studied polymorphisms, subjects homozygous for the minor alleles (n=3 for rs8102595, n=16 for rs3826795) were combined with heterozygous groups (i.e. dominant mode of inheritance was used statistical analyses).

p-value adjusted for age, gender and BMI and diabetes status; BMI – Body Mass Index, WHR – waist-to-hip ratio, SAT – subcutaneous adipose tissue, CRP – C-reactive protein, IL-6 – Interleukin 6, HbA1c – Glycohemoglobin, oGTT – oral Glucose Tolerance Test, FPG – Fasting plasma glucose, FPI – Fasting plasma insulin, GIR – Glucose infusion rate during the steady state of an euglycemic hyperinsulinemic clamp, HDL-C – high Density Lipoprotein Cholesterol, LDL-C – Low Density Lipoprotein Cholesterol , FFA – Free Fatty Acids, TG – Triglycerides, ALAT – alanine aminotransferase, ASAT - aspartate aminotransferase, gGT -Gamma-glutamyl transferase, TSH – thyroid-stimulation hormone, fT3 – free triiodothyronine, fT4 – free tetraiodothyronine, Met Blood (%)/ Met Sc (%) / Met Visc (%) - Methylation of cg22891070 in *HIF3A* in blood / SAT / VAT, *HIF3a* mRNA – mRNA expression of *HIF3A* in subcutaneous/visceral adipose tissue.


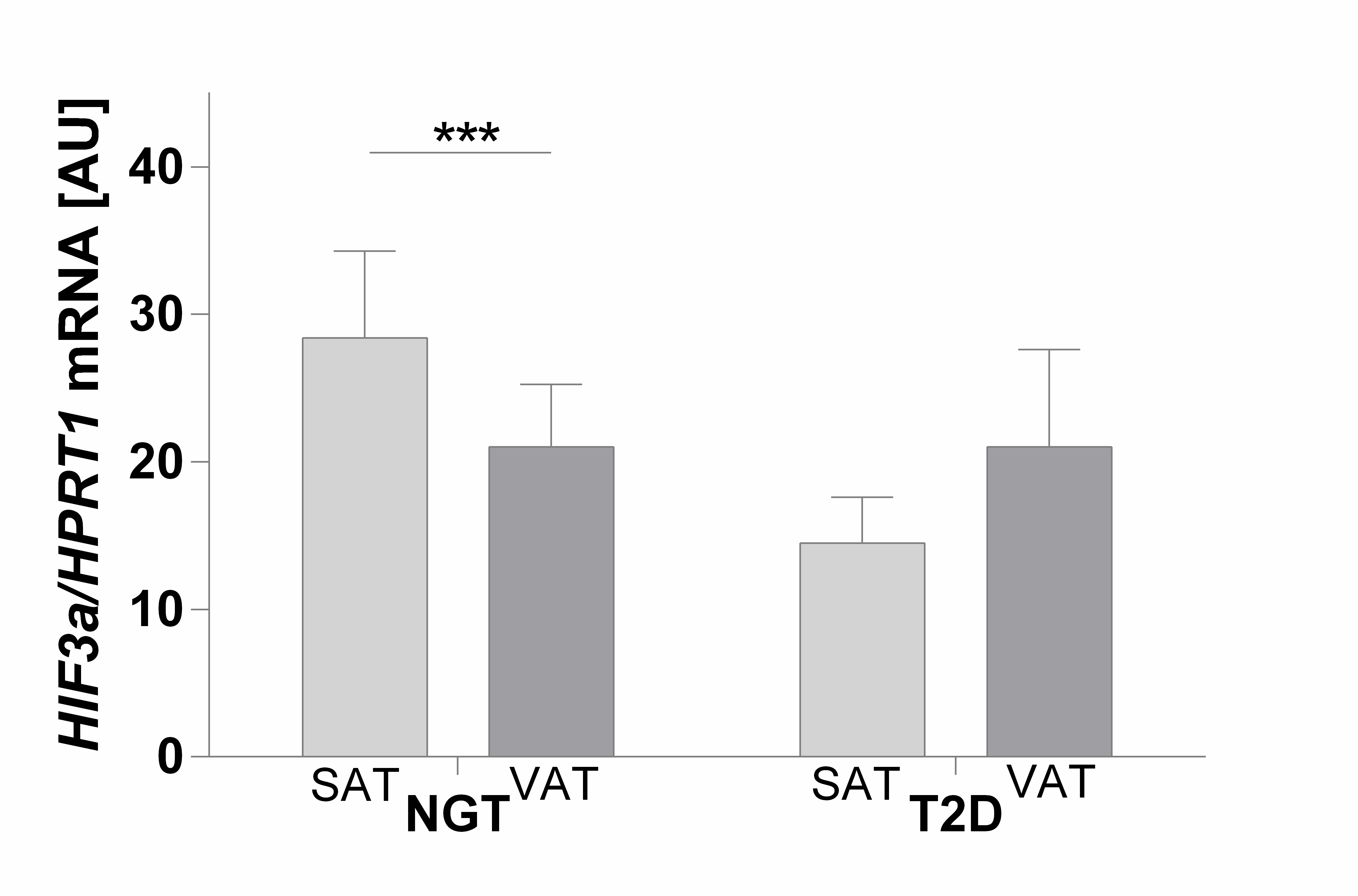


**Supplementary Figure:** Fat depot-related *HIF3A* mRNA expression pattern is distinct in individuals with either normal glucose tolerance (NGT; SAT, n=316; VAT, n=242) or type 2 diabetes (T2D; SAT, n=318; VAT, n=245)
